# Green Tea (*Camellia sinensis*): A Review of Its Phytochemistry, Pharmacology, and Toxicology

**DOI:** 10.3390/molecules27123909

**Published:** 2022-06-18

**Authors:** Tiantian Zhao, Chao Li, Shuai Wang, Xinqiang Song

**Affiliations:** 1 School of Medicine, Xinyang Normal University, Xinyang 464000, China; ttzhao2020@163.com (T.Z.); ws20095072016@163.com (S.W.); 2 College of Chemistry and Chemical Engineering, Xinyang Normal University, Xinyang 464000, China; lichaoxynu@163.com

**Keywords:** green tea, unfermented tea, phytochemistry, pharmacology, toxicology, human health

## Abstract

Objectives Green tea (*Camellia sinensis*) is a kind of unfermented tea that retains the natural substance in fresh leaves to a great extent. It is regarded as the second most popular drink in the world besides water. In this paper, the phytochemistry, pharmacology, and toxicology of green tea are reviewed systematically and comprehensively. Key findings Green tea has been demonstrated to be good for human health. Nowadays, multiple pharmacologically active components have been isolated and identified from green tea, including tea polyphenols, alkaloids, amino acids, polysaccharides, and volatile components. Recent studies have demonstrated that green tea shows versatile pharmacological activities, such as antioxidant, anticancer, hypoglycemic, antibacterial, antiviral, and neuroprotective. Studies on the toxic effects of green tea extract and its main ingredients have also raised concerns including hepatotoxicity and DNA damage. Summary Green tea can be used to assist the treatment of diabetes, Alzheimer’s disease, oral cancer, and dermatitis. Consequently, green tea has shown promising practical prospects in health care and disease prevention.

## 1. Introduction

Tea has a long history, which originates from China and spreads all over the world by direct or indirect ways. Nowadays, tea has been consumed by 3 billion people worldwide, which is considered as one of the most popular non-alcoholic beverages [1]. Tea can be classified in many types according to the diverse definition methods in different countries. In China, according to the degree of fermentation, tea is divided into six major tea lines: green tea, black tea, white tea, yellow tea, Oolong tea, and dark tea [2]. Green tea was the first tea to be discovered, and it is a non-fermented tea. Green tea retains more natural substances in fresh leaves and has less vitamin loss, thus forming the characteristics of green tea as “clear soup with green leaves and strong flavor convergence”. The main varieties of green tea are *Longjing*, *Biluochun*, *Huangshanmaofeng*, *Xinyangmaojian*, etc.

A large number of researchers have confirmed that green tea possesses chemical ingredients that are closely related to human health. Tea polyphenols, caffeine, theanine, tea polysaccharides, and other components which are extracted and separated from green tea have pharmacological activities such as anti-cancer [3], anti-oxidation [4], protecting the nervous system [5], and lowering blood sugar [6]. Green tea has been considered to be suitable for patients with hypertension, hyperlipidemia, coronary heart disease, arteriosclerosis, and diabetes. However, it is important to keep in mind that “natural” does not mean perfectly safe. Although the toxic side effects of green tea are relatively small, it must be used with caution in pregnancy, children, and the elderly population.

Tea polyphenols are one of the main components in the formation of the color and flavor of tea soup and are also important ingredients for tea with health functions [7]. The species, processing method, and fermentation degree are the key factors that affect the content of tea polyphenols in tea [8]. Gao et al. analyzed the content of 16 common tea leaves and found that the content of tea polyphenols in green tea was the highest [9]. They suggested that green tea was the preferred tea source for the development of tea polyphenol functional foods [9]. 

In recent years, numerous domestic and foreign studies are focused on the chemical composition and pharmacological effects of green tea. At present, there is a lack of systematic and comprehensive review on the research results of green tea. In this paper, we review the phytochemistry, pharmacological activity, and toxicology of green tea, with the purpose of promoting further research on green tea and developing the precious green tea resources in China. 

## 2. Methods

The data were collected by searching PubMed, Google Scholar, Web of Science, and CNKI. The keywords used as search terms were green tea, phytochemical, chemical composition, EGCG, pharmacology, tea polyphenols, antioxidant, cancer, diabetes, antibacterial, antiviral, AD, PD, immune T cells, and toxicology. Various related articles and websites were also included. For further research, the references of some selected articles were also searched. The inclusion criteria for this review were systematic reviews and experimental studies on green tea. However, studies on other types of tea such as yellow tea, dark tea, or other natural plants were considered ineligible for inclusion. In addition, other types of articles, such as conference reports, case reports, and short communications, were also excluded. No time limitation was considered in this review. 

## 3. Phytochemistry

Tea is rich in healthcare ingredients and pharmacologically active ingredients. From the beginning of the 19th century to the present, it has been reported that more than 500 chemical components have been isolated from tea, including more than 400 organic compounds and more than 40 inorganic compounds [10]. Green tea, as a non-fermented tea, retains the original chemical components of tea completely. This section details the research on green tea in phytochemistry and classifies the main compounds in green tea. The chemical structures of the main compounds that have been identified are listed in the figures below.

### 3.1. Tea Polyphenols

Tea polyphenols is a general term for polyphenols in tea. There are about 30 kinds of compounds, mainly composed of catechins, flavonoids, anthocyanins, and phenolic acids [11]. The highest content of tea polyphenols in green tea is 20–30%, which can be used as an excellent natural antioxidant. 

#### 3.1.1. Catechins (**1**–**5**) 

**Figure 1:**
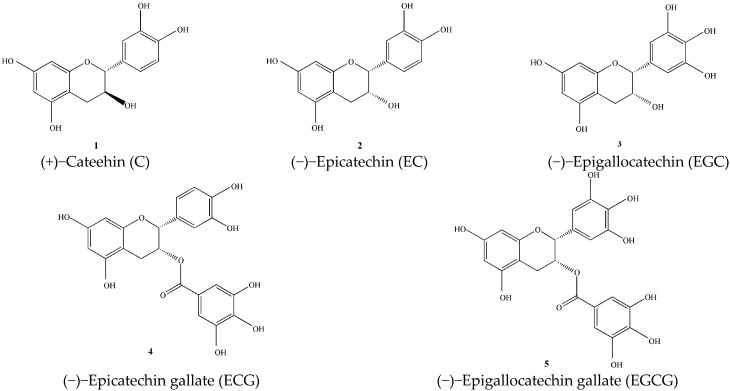
Chemical structures of catechins isolated from green tea.

The catechins in tea mainly include catechin (C), epicatechin (EC), epigallocatechin (EGC), epicatechin gallate (ECG), and epigallocatechin Gallate (EGCG) [12]. As shown in Figure 1. A large number of studies have shown that catechins in green tea, especially EGCG, have anti-cancer [13], anti-viral [14], and anti-oxidant effects [4].

#### 3.1.2. Flavonoids (**6**–**25**)

**Figure 2:**
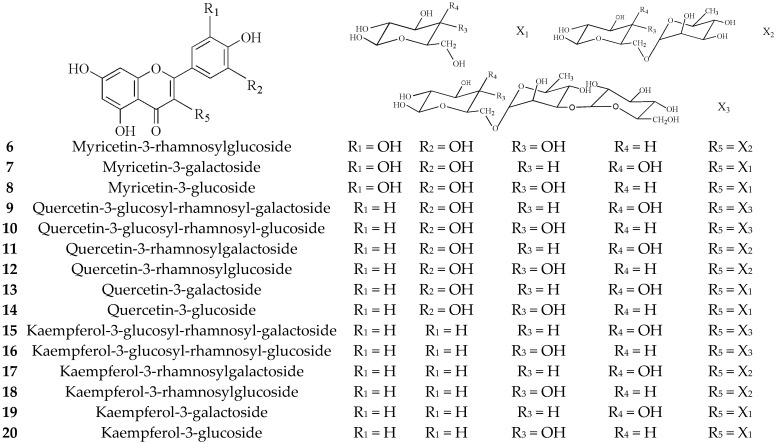
Chemical structures of flavonoids isolated from green tea.

Green tea is rich in flavonol glycosides, mainly including myricetin glycosides, quercetin glycosides, and behenyl glycosides [15,16]. This sugar chain consists of monosaccharides, such as glucose, galactose, rhamnose, arabinose, etc., and disaccharides or trisaccharides [17], as shown in Figure 2.

**Figure 3:**
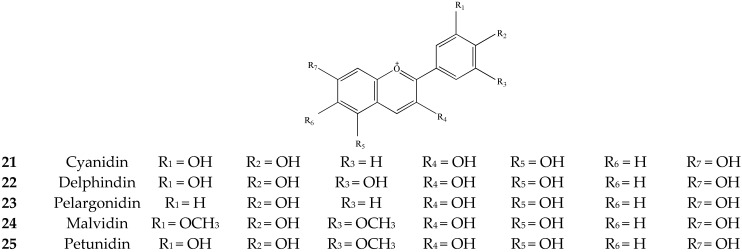
Chemical structures of anthocyanins isolated from green tea.

Anthocyanins are a class of water-soluble pigments and belong to flavonoids [18]. The content of anthocyanins is not high in tea, but due to its obvious bitter taste, it has a great impact on tea quality [19]. As presented in Figure 3.

#### 3.1.3. Phenolic Acids (**26**–**31**)

**Figure 4:**
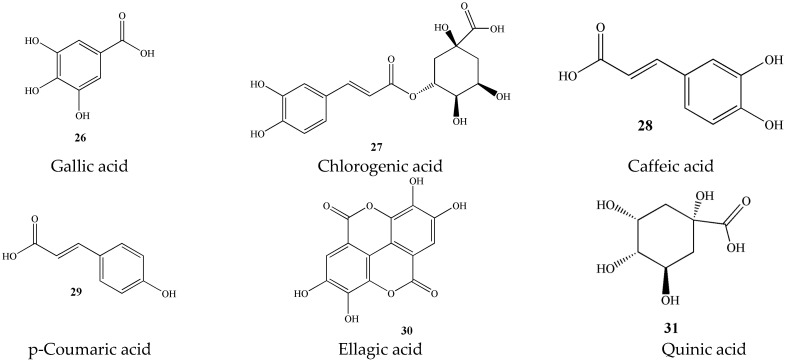
Chemical structures of phenolic acids isolated from green tea.

At present, there are few studies on phenolic acid compounds in green tea. The content of phenolic acids in green tea is relatively small, but it includes various ingredients such as gallic acid, chlorogenic acid, caffeic acid, p-coumaric acid, ellagic acid, quinic acid, and tea gallate [20]. As presented in Figure 4.

### 3.2. Alkaloids (***32***–***34***)

**Figure 5:**
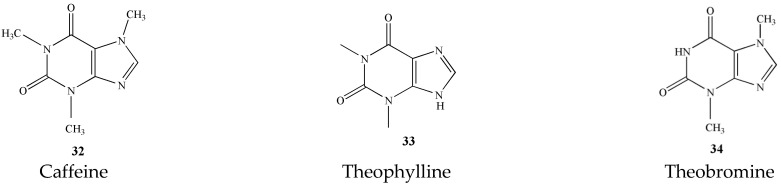
Chemical structures of alkaloids isolated from green tea.

The alkaloids in tea are mainly purine alkaloids. Among them, the caffeine content is the most (2~5%). Secondly, it also contains a small amount of theophylline and theobromine [21]. These three alkaloids are the main material basis for the refreshing effect of tea [22]. The names and structures of these three alkaloids are detailed in Figure 5.

### 3.3. Amino Acids (***35***–***39***)

**Figure 6:**
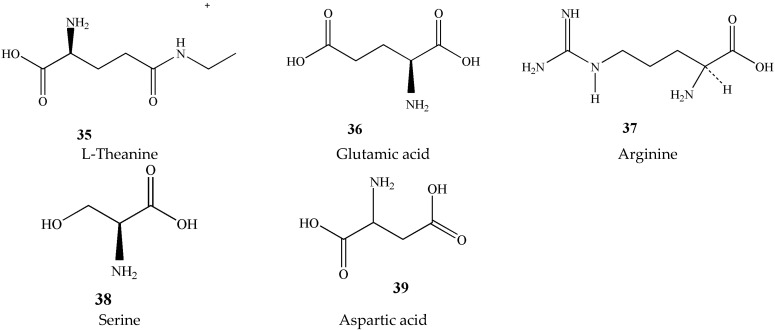
Chemical structures of amino acids isolated from green tea.

The type and content of amino acids in tea is one of the most important substances affecting tea quality. Tea contains about 1% to 4% of amino acids. So far, 26 amino acids have been found in tea, including 20 protein amino acids and 6 non-protein amino acids. The highest content is theanine, glutamic acid, arginine, serine, and aspartic acid, shown in Figure 6 [23]. Theanine and γ-aminobutyric acid are two important active amino acids in tea. They have notable protective effects on the nervous system [24,25]. Theanine accounts for approximately 50% of all amino acids; however, γ-aminobutyric acid is low. Chen et al. used an amino acid analyzer to determine the content of free amino acids in several different teas and found that there was no significant difference in the amino acid composition of green tea and black tea [26].

### 3.4. Carbohydrate

The reason why the tea soup is slightly sweet is that tea contains a small amount of monosaccharides and disaccharides, such as glucose, fructose, galactose, sucrose, etc. Most carbohydrates in tea are polysaccharides, such as cellulose, starch, and pectin, which are insoluble in water [11]. 

### 3.5. Aromatic Ingredients (***40***–***56***)

**Figure 7:**
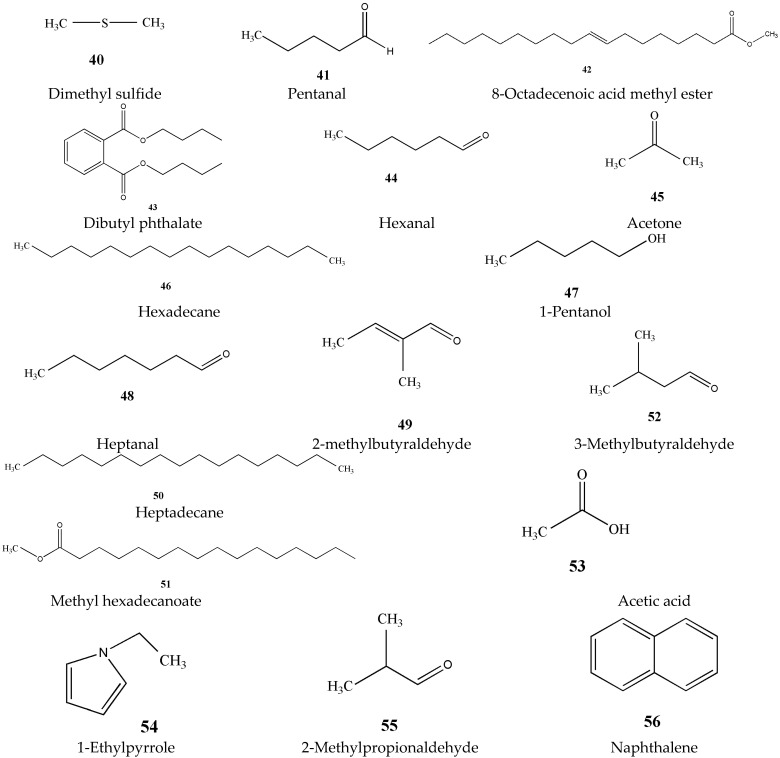
Chemical structures of aromatic ingredients isolated from green tea.

The substances that form the aroma of green tea are mainly volatile aromatic substances. Among the chemical components of green tea, the aroma components do not occupy much content, about 0.005% to 0.020%, but the types are quite complicated [27]. There have been many reports on the analysis of volatile components in green tea, and at the same time, new components have been discovered and identified. The main aroma components of green tea are listed in Figure 7.

### 3.6. Organic Acids (***57***–***65***)

**Figure 8:**
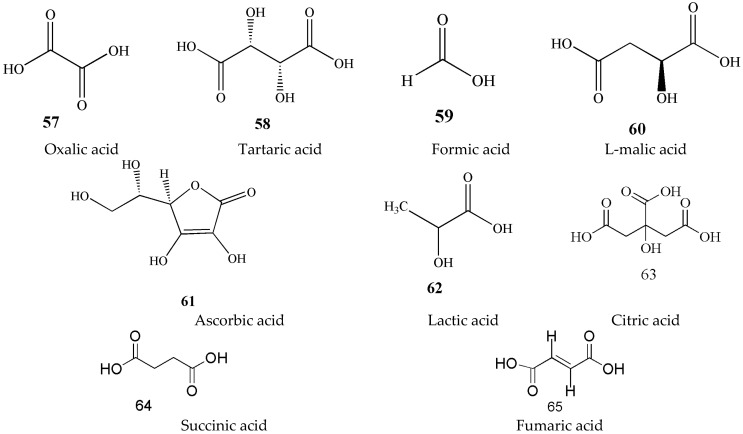
Chemical structures of organic acids isolated from green tea.

Organic acids in green tea, as a water-soluble substance, are one of the main components that affect the aroma and taste of tea soup [28,29]. More than 40 organic acids have been isolated and identified from tea, including free organic acids in tea soup and more than 30 in aroma components [30]. Volatile compounds such as acetic acid, butyric acid, and hexenoic acid are classified under the aromatic substance category. Therefore, this section only describes the non-volatile organic acids in Figure 8.

### 3.7. Mineral Elements

The inorganic compounds in tea are called ash, which is mainly composed of some mineral elements and their oxides. Ash content is one of the indexes for quality inspection of tea export. The most abundant mineral elements are P and K, followed by Ca, Mg, Fe, Mn, Al, S, Si, and trace elements such as Zn, Cu, and F [31,32,33]. Due to the valuable significance of mineral elements on the physiological function of tea plant and human body, it has aroused extensive attention of scientists. 

### 3.8. Others

In addition to the chemical components mentioned above, green tea also contains a certain amount of vitamins, such as vitamin B, vitamin C, and vitamin E [34]; enzymes, such as glucosidases and lipoxidases [35]; and chlorophyll, which is a highly safe natural edible pigment [36].

## 4. Pharmacology

### 4.1. Antioxidant Effects

As early as 1997, it was reported that green tea extract and its three main components, including tea polyphenols, theanine, and caffeine, have the ability to effectively inhibit copper-catalyzed low-density lipoprotein (LDL) lipid peroxidation. Moreover, Yokozawa T et al. found that the antioxidant activity of green tea extract was in a dose-dependent manner, and the antioxidant activity of the three components was tea polyphenols > theanine > caffeine. Finally, they came to the conclusion that chelated metal ion copper is considered to be one of the possible mechanisms of green tea against peroxidation [37]. In the current study, the antioxidant activity of green tea could be studied by two different chemical tests. (1) DPPH free radical scavenging test. Sun et al. (2007) demonstrated that the anti-free radical ability of antioxidant compounds contained in green tea was ranked as EGCG > ECG > EGC > EC. In 2020, one study showed that the ethanolic extract of green tea has powerful antioxidant activity by DPPH testing with low IC_50_ values of 0.005 μg/mL [38]. (2) Total oxy-radical scavenging capacity (TOSC) assay [39]. Kang et al. (2010) revealed that ECG and EGCG have been proven to have the greatest antioxidant effects, followed by EC, C, GA, and EGC [40]. In summary, the results of the two test methods were consistent. 

In recent years, there is no doubt that the antioxidant effects of tea polyphenols and catechins in green tea have been extensively studied by scholars all over the world. However, little research has been performed on antioxidants in green tea polysaccharides. Wang et al. (2012) reported that tea leaves polysaccharides (TLPS), tea seed polysaccharides (TSPS), and tea flower polysaccharides (TFPS) isolated from tea leaves, flowers, and seeds, at concentrations ranging from 0.5~100 μg/mL, could exhibit a dose-dependent superoxide scavenging activity. In particular, at a concentration of 400 μg/mL, the superoxide radical scavenging rates of TLPS, TFPS and TSPS were as high as 90.45%, 78.58%, and 58.34%, respectively [41].

In addition to this, Hsu et al. (2014) suggested that the green tea extract (125, 625, and 1250 mg/kg, i.g., for four weeks) showed notable protective effects on redox imbalance in the brain of aging mice, and this mechanism may be associated with more efficient clearance of ROS by increasing the activity of antioxidant enzymes, such as superoxide dismutase (SOD), catalase, glutathione peroxidase (GSH-Px), and Glutathione reductase (GSH-Rd) in the brain [42]. In one report of 2018, it was reported that the antioxidant activity of green tea extract could protect against hepatotoxicity caused by excess acetaminophen (APAP) in mice [43].

### 4.2. Anticancer Effects

It is reported that green tea has a therapeutic effect on various cancer types [44,45]. In recent years, research on the anticancer mechanism of green tea has attracted more and more attention. It is mainly reflected in the following points.

(1) Inhibiting migration and invasion of tumor cells. In 2012, an interesting experiment showed that EGCG inhibited the growth of HeLa cells in a dose- and time-dependent manner. In particular, the IC_50_ values of cell viability of HeLa cells at concentrations of 50 μM and 100 μM were 57.2% and 29.3% at 48 h, respectively. In addition, Sharma et al. revealed that this mechanism may be that EGCG could effectively inhibit the invasion and migration of HeLa cells and regulate the expression of MMP-9 and TIMP-1 [46]. Luo et al. (2014) reported for the first time that green tea extract possessed an ability to significantly inhibit lung and liver metastasis in BALB/c mice with 4T1 tumors with reduced abilities of 54.5% and 72.6%, respectively [47].
(2) Inducing apoptosis. Cerezo-Guisado et al. (2015) performed cytotoxic activity tests on EGCG, a major component of green tea. The results showed that the mortality of HT-29 cells could reach 100% after treatment with 100 or 200 μM EGCG. In addition, they revealed that EGCG could induce apoptosis in colon cancer cells by modulating Akt, ERK1/2 and p38 MAPK signaling pathways [48]. Roychoudhury et al. (2018) treated pig ovarian granulosa cells with five different doses of green tea extract, and evaluated the hormone released by granulosa cells by EIA. It was found that at the highest dose (200 μg/mL), the apoptosis markers caspase-3 and p53 were increased in granulosa cells. Therefore, they suggested that activation of caspase-3 and p53 could ultimately induce apoptosis in ovarian cells [49].
(3) Inhibiting tumor cell angiogenesis. In vitro, EGCG displayed growth inhibition on HuH7 cells and Hc cells with IC_50_ values of 25 μg/mL and 84 μg/mL, respectively. Further investigations revealed that the mechanism of these effects may be associated with the inhibition of VEGF binding to receptor tyrosine kinases and decreased expression of Bcl-xL protein and VEGF mRNA [50].
(4) Inhibiting the proliferation of tumor cells. Studies have found that EGCG could inhibit the growth of androgen-sensitive human prostate cancer cells (PCA) in a dose-dependent manner, and this effect may be mediated by G_0_/G_1_ phase cell cycle arrest caused by WAF1/p21 [51]. Ma et al. (2013) reported that EGCG could effectively inhibit the growth of gastric cancer cell line NCI-N87 in a time- and dose-dependent manner. At the same time, they found that the new mechanism in the treatment of gastric cancer could be that EGCG could increase the expression of KLF4, change the expression of p21, CDK4 and cyclin D1, and then arrest the cell cycle in G_0_/G_1_ phase [52].
(5) Other mechanisms. 4-NQO has been confirmed to induce a variety of cancers [53]. In one report of 2008, Srinivasan et al. established a 4-NQO-induced rat oral cancer model to study the therapeutic effect of green tea polyphenols on oral cancer. After treatment with 200 mg/kg of green tea polyphenols, the number of tumors, tumor volume, and oral squamous cell carcinoma were significantly decreased by 66.27%, 56.80%, and 88.75%, respectively. In addition, they also suggested that GTP acted as a detoxifier here, which in turn inactivated carcinogens [54]. In 2012, Lu et al. revealed that green tea extract could up-regulate the expression of ANX1, an important anti-inflammatory mediator [55], in human non-small cell lung cancer cell lines, and down-regulate the expression of COX-2. Therefore, they proposed that the new mechanism of green tea extract to prevent lung cancer may be that green tea polyphenols could target a variety of inflammatory pathways to induce tumor cell apoptosis [56].

### 4.3. Anti-Diabetic Effects

Anti-diabetic effect is one of the important biological activities of green tea. Current studies have shown that the anti-diabetic effect of green tea is mainly achieved through the following four mechanisms.

(1) Improving insulin resistance. The endocrine function of adipocytes plays a central role in insulin resistance [57]. Wu et al. found that green tea polyphenols could increase insulin sensitivity in rats by increasing the absorption of glucose by adipocytes and their ability to bind to insulin [58]. Membrane transport of insulin-regulated glucose transport protein (GLUT-4) is critical for maintaining blood glucose balance in the body [59]. Serisier et al. found that green tea extract (80 mg/kg, i.g., for 12 weeks) could improve insulin sensitivity and lipid distribution by altering the expression of genes involved in glucose and lipid homeostasis, including GLUT-4, LPL, and PPAR, which ultimately led to a decrease in blood glucose and an improvement in insulin resistance in obese dogs [60].
(2) Improving glucose metabolism. Sundaram et al. (2013) revealed that green tea extract (75 mg/kg, i.g., for 30 days) had significant hypoglycemic effects on streptozotocin-induced diabetic rats. Moreover, its ability to lower blood sugar was comparable to the oral hypoglycemic drug metformin. The mechanism of this action was related to the increase of glycogen content in the liver and the change of the activity of key enzymes in glucose metabolism [61].
(3) Promoting insulin secretion. Wang et al. isolated the water-soluble polysaccharide 7WA from the leaves of green tea and studied its anti-diabetic effect in 2015. They found that 7WA could promote insulin secretion and had a significant hypoglycemic effect through a possible mechanism of cAMP-PKA dependent pathways [62].
(4) Improving diabetic complications. Impaired cardiac function in diabetes is closely related to hyperglycemia [63,64]. Green tea is rich in polyphenol antioxidants, which can effectively prevent heart disease [65]. Babu et al. found that green tea extract (300 mg/kg, i.g., for four weeks) could significantly reduce the blood sugar, lipid peroxide, the levels of triglyceride, and the degree of protein glycosylation in the heart of diabetic rats. In 2016, Zhong et al. demonstrated that EGCG treatment with 10 µM showed significant inhibiting effects on neural tube defects in diabetic pregnant mice with the defect rate decreased from 29.5% to 2%. The underlying mechanism for this effect might be related to that 10 μM EGCG could inhibit the hypermethylation of DNA by blocking the increased expression and activity of DNA methyltransferase in maternal diabetic mice [66].

### 4.4. Antibacterial Effects

It is well known that green tea exhibits antibacterial effects against a variety of bacteria. In 2000, Yee et al. first reported that green tea had the ability to inhibit *Helicobacter pylori activity*. Their studies have shown that EGCG, and EC could inhibit the growth of *Helicobacter pylori* with MIC_90_ values of 50~100 μg/mL, and 800~1600 μg/mL, respectively. The results indicated that EGCG might be the most effective component against *Helicobacter pylori* activity [67]. In one report of 2006, Anand et al. found that EGCG could down-regulate TACO gene expression in a dose-dependent manner by flow cytometry experiments to inhibit the survival of *Mycobacterium tuberculosis* in macrophages. Moreover, they also proposed that EGCG has the potential to be an effective drug for the treatment of tuberculosis [68]. As early as 1995, some scholars have found that green tea extract could effectively inhibit the growth of major foodborne pathogens, including *E. coli*, *Staphylococcus aureus*, *Salmonella typhimurium*, *Listeria monocytogenes*, and so on [69]. However, it is unclear what the main antibacterial ingredients in green tea extracts are, and which bacteria are most strongly inhibited. In 2006, Si et al. used HSCCC technology to isolate epicatechin gallate (ECG), epigallocatechin gallate (EGCG), epicatechin (EC) and caffeine (CN) from green tea extracts and further compared their antibacterial activity. Then, it was found that EGCG had the highest antibacterial activity, especially the most significant inhibitory effect on *S. aureus* with the MIC_90_ value of 58 mg/L [70]. Moreover, Sharma et al. (2012) revealed that green tea extracts showed significant antibacterial activity against skin pathogens in vitro, and this mechanism was mainly related to preventing bacterial adhesion [71].

Some studies have shown that drinking green tea was beneficial to oral health [72,73]. Moreover, Fournier-Larente J et al. further studied the effect of green tea on periodontal pathogens. In vitro, both green tea extract and EGCG exhibited significant antibacterial effects on *Porphyromonas gingivalis* with MIC values ranging from 250 to 1000 mg/mL, 125 to 500 mg/mL, respectively. This mechanism for effect was related to reducing the adhesion to *P. gingivalis* and regulating the gene expression of *P. gingivalis* [74]. These findings showed that green tea could be regarded as a natural treatment for periodontitis. In 2019, Ignasimuthu K et al. revealed that the inhibitory effect of green tea on common food-borne pathogens was enhanced by increasing its lipophilicity [75].

### 4.5. Antiviral Effects

In 2002, it was reported that when the concentration of EGCG was greater than 1 μM, EGCG destroyed virus particles and significantly inhibited post-adsorption entry and reverse transcription in acutely infected monocytes. In addition, above 10 μM, protease kinetics was also inhibited. At the same time, they found that the inhibitory effect of EGCG modified by liposomes was significantly improved [76]. In addition, it has been reported that catechin in green tea could reversibly inhibit HIV-1 integrase activity [77]. In 1993, there were studies have shown that EGCG could affect the infectious capacity of influenza viruses by agglutinating the virus [78]. Song et al. (2005) found that the order of antiviral activity of green tea polyphenols was EGCG > ECG > EGC. Among them, EGCG had the strongest activity, and its EC_50_ value for influenza A virus was 22~28 μM [79]. The World Health Organization (WHO) considers hepatitis B to be the world’s most important public health problem. Xu et al. (2008) revealed that green tea extract (GTE) could significantly inhibit the production of hepatitis B virus (HBV), and pointed out that the efficacy of EGCG was relatively weak compared with GTE. However, the exact mechanism of GTE anti-HBV needs to be further elucidated [80].

Apart from these, foodborne viruses, such as the human Novo virus (Nov) and hepatitis A virus (HAV), are considered to be the major pathogens of food-borne diseases. Randazzo W et al. suggested that green tea extract (10 mg/mL, for 30 min) could cause MNV and HAV to be completely inactivated in the surface disinfection test. The results showed that green tea extract could be used as a natural disinfectant to improve food safety and quality [81]. Zika virus (ZIKV), transmitted by mosquito bites, could cause a variety of neurological diseases [82]. Sharma et al. (2017) initially explained that this potential mechanism by which EGCG inhibited ZIKV entry into host cells might be related to the binding of envelope proteins [83]. 

Since the rapid spread of Corona Virus Disease 2019 (COVID-19), due to the shortage of antiviral medicines, finding alternative medicines from natural herbs to prevent COVID-19 is considered a safe strategy to prevent the pandemic. Mhatre S et al. reported and summarized the research of EGCG in anti-COVID-19 virus in detail, and then proposed that EGCG can be used as a dietary supplement or functional food for the prevention and treatment of COVID-19 [84]. Whether EGCG has a synergistic effect on a COVID-19 vaccine is the focus of future research by discussing the multiple antiviral inhibitory effects of EGCG [85].

### 4.6. Neuroprotective Effects

Green tea is rich in polyphenols [86]. The brain permeability of polyphenols makes it an important class of drugs for the treatment of neurodegenerative diseases. There are studies have shown that green tea polyphenols play an important role in the treatment of neurodegenerative diseases. The protective effect on the nervous system is mainly reflected in the following aspects.

(1) Effect on Alzheimer’s disease (AD). Alzheimer’s disease is characterized by memory and other cognitive declines. In 2008, Kaur et al. showed that green tea extract (0.5%, i.g., for 8 weeks) could significantly improve learning and memory in aged rats, and they also found that green tea extract had selective inhibition of acetylcholinesterase. It was reported that green tea catechins could promote cognitive dysfunction in AD model rats through the antioxidant defense, but the exact defense mechanism still needs to be further explored [87]. In one report of 2018, L-theanine could improve memory and hippocampal LTP in AD mice. This effect may be related to the regulation of hippocampal synaptic efficacy through the dopamine D1/5-PKA pathway. Moreover, they proposed a point that L-theanine could be a candidate drug for AD treatment [88]. An interesting study has shown that green tea had better neuroprotective effects than black tea on memory deficit and hippocampal oxidation status in AD mice [89].
(2) Effect on Parkinson’s disease (PD). EGCG, a major active ingredient of green tea polyphenols, was a natural iron chelator that had a neuroprotective effect on neurotoxins in mice and rats [90]. Both NO and reactive oxygen species (NOS) are involved in the pathogenesis of neurological diseases such as PD [91]. Guo et al. used various techniques, such as immunohistochemistry, to reveal that green tea polyphenols (GTP) could protect dopamine neurons in 6-OHDA-treated PD rat models through a pathway related to inhibition of NO and reactive oxygen species (ROS) [92]. In one recent study, green tea polyphenols (GTP) possessed an ability to significantly improve nerve redox imbalance and mitochondrial dysfunction by regulating circadian rhythm [93].
(3) Effect on cerebral ischemia. The overexpression of MMPs is closely related to the pathological process of focal cerebral ischemia [94]. In 2009, Park et al. suggested that EGCG at a dose of 50 mg/kg could reduce neuronal damage after cerebral ischemia. This potential mechanism may be related to the inhibition of MMP-9 activity [95]. In addition, it has been reported that 400 mg/kg of green tea polyphenols could improve the spatial cognitive ability after chronic cerebral hypoperfusion by scavenging oxygen free radicals, reducing the production of lipid peroxides and reducing the damage of oxidized DNA, thereby playing a neuroprotective role [96]. Theanine is easily involved in various neurophysiological activities through the blood–brain barrier, and the neuroprotective effect of theanine on brain damage caused by cerebral ischemia has recently been reported [97]. In 2020, Zhao et al. studied the exact molecular mechanism of the protective effect of cerebral ischemia/reperfusion (IR) injury and found that theanine had a protective effect on hippocampal injury in IR rats. This mechanism may also be related to inhibition of HO-1 expression and activation of the ERK1/2 reperfusion injury pathway, in addition to inhibition of oxidative stress [98].
(4) Effect on brain injury. Some pesticides have been confirmed to be an environmental toxin that causes degeneration of dopaminergic neurons [99]. Tai et al. reported that EGCG had a protective effect on DDT-induced dopaminergic cell death [100]. It is well known that heavy metal poisoning, especially lead, can cause nervous system damage [101]. In addition, 5 μg/L green tea extract had effective protection against lead-induced brain oxidation and DNA damage in rats [102]. It is worth noting that narcotic drugs have a certain effect on the damage of the nervous system [103,104]. In one report of 2019, green tea polyphenols (GTP) (25 mg/kg, i.g., for 7 days) could improve cognitive impairment caused by isoflurane by regulating oxidative stress [105].

### 4.7. Effects on the Immune System

To date, there has been the little pharmacological study of green tea on the immune system. EGCG (2.5 μM~10 μM) could inhibit the proliferation of spleen T cells in C57BL mice in a dose-dependent manner. This mechanism may be related to the inhibition of IL-2/IL-2 receptor signaling [106]. Other research data indicated that EGCG could induce Foxp3 expression through a novel epigenetic mechanism, thereby inducing regulatory T cells [107]. In 2014, Balaji J et al. found that green tea water extract can significantly reduce the mortality of mice with anaphylactic shock induced by compound C48/80. This finding provided experimental support for green tea in the treatment of asthma and allergic rhinitis [108]. Wu et al. also reported that EGCG showed positive effects on experimental autoimmune encephalomyelitis (EAE) mice by inhibiting T cell proliferation [109].

### 4.8. Other Pharmacological Effects

In addition to the pharmacological effects summarized above, green tea also possesses several other aspects of pharmacological activity. In 2004, Geetha T et al. explored the relationship between the antioxidant activity of green tea polyphenols and their antimutagenicity through different in vitro antioxidant methods and the highly standardized Ames microsomal test system. Finally, it was found that its in vitro antioxidant activity was closely related to its anti-mutagenic effect [110]. In 2005, Santhosh KT et al. first reported that green tea polyphenols inhibited the mutagenicity of tobacco in a concentration-dependent manner through its inhibition of nitrosation [111]. Apart from these, in in vivo experiments, it was observed that thyroid peroxidase and 50-deiodinase I activities were reduced in male albinism rats after treatment with high doses of green tea and catechins. These results indicated that catechins in green tea extract may play an antithyroid role [112]. 

As we all know, the prevalence of hypertension is increasing year by year in every country in the world. Diuretics, especially hydrochlorothiazide (HCTZ), are widely used in the treatment of hypertensive patients with ischemic heart disease. At present, studies have shown that the significant incidence of diuretic-induced hypokalemia with ventricular ectopic activity is a serious problem in clinical medical treatment [113,114]. Chakraborty et al. were the first to study the effects of green tea alone and in combination with hydrochlorothiazide (HCTZ) on diuretic activity. The results showed that green tea extract at doses of 100 and 500 mg/kg showed significant diuretic activity; furthermore, green tea extract also significantly reduced potassium loss in the combination group compared with the HCTZ-treated group alone [115]. Susilowati et al. (2019) also reported that green tea extract at a dose of 70 mg/kg had diuretic activity equivalent to furosemide, which may be related to the increase of glomerular filtration rate by increasing blood flow and cardiac output to the kidneys [116].

An in vivo experiment in rats demonstrated that green tea polyphenols have beneficial effects on the bone mineral density of cancellous and cortical bone compartments in ovariectomized rats [117]. Recently, Khademvatan S et al. demonstrated that green tea compounds had anti-protozoal Leishmania effects through bioinformatics analysis [118].

**Figure 9:**
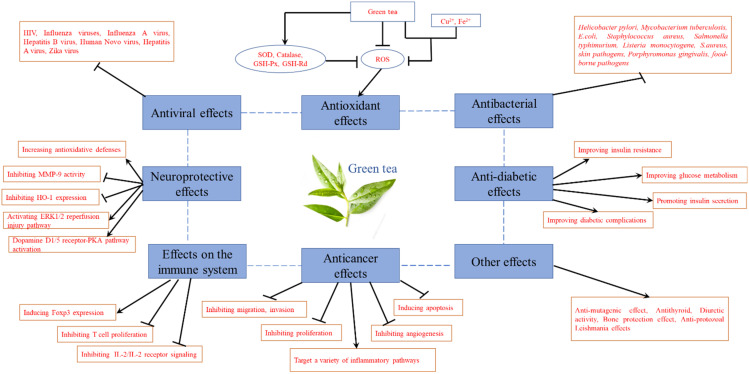
Pharmacological effects of green tea.

Therefore, the pharmacological activities of green tea were summarized as shown in Table 1 and Figure 9.

## 5. Toxicology

Green tea is a popular beverage, especially in China and Japan. There are no reports of clinical toxicity on whether there is a health hazard in drinking a large amount of green tea every day. In 2008, Chengelis et al. first conducted safety studies on standardized green tea catechin (GTC) preparations, as well as heat-sterilized (GTC-H) and non-heat-sterilized (GTC-UH) preparations, and found that the level of no observed adverse effects of the three preparations (NOAEL) was 2000 mg/kg/day [119]. Another study demonstrated that GTC-H did not affect embryonic development in female rats. Its NOAEL was also 2000 mg/kg/d [120]. Furthermore, Hsu et al. used a subacute exposure paradigm to evaluate that green tea extract (2500 mg/kg, i.g., for 28 days) would not cause death or toxicity in ICR mice [121]. These valuable data provide the basis for the safe application of green tea extract in food production.

Green tea is often developed as a weight-loss beverage, and they are usually considered as safe. However, the United States Pharmacopoeia (USP) has conducted a safety review and counted adverse events (AEs) after the use of high-dose GTE preparations, most of which were liver injury reports [122,123,124,125]. In 2010, in an in vivo experiment, Lambert et al. reported for the first time that plasma alanine aminotransferase (ALT) in male CF-1 mice increased 138 times after treatment with high-dose EGCG (1500 mg/kg, i.g., for 7 days). Therefore, they came to the conclusion that high doses of EGCG had a hepatotoxic effect. This mechanism of toxicity might be related to the induction of oxidative stress in the liver. However, the observed toxic doses were much higher than normal tea consumption [126]. In 2018, Hu et al. evaluated the safe dosage of green tea for adults based on toxicological data and AEs. The safe intake for adults should be controlled below 338 mg EGCG/day [127].

Furukawa et al. investigated whether EGCG could cause oxidative damage to in vitro bovine thymus DNA under the action of metal ions and H_2_O_2_ oxidative stress. They observed that EGCG promoted the formation of 8-oxide, a characteristic oxidative damage to DNA that is strongly associated with mutations and cancer [128]. Therefore, they came to the conclusion that that this oxidative damage to EGCG could be considered as a potential predisposing factor for EGCG carcinogenicity [129]. One study showed that EGCG (20, 40 and 80 μM, 10, 60, and 240 min) caused DNA damage in both human lymphocytes and Nalm6 cells in a dose-dependent manner. When the maximum dose of EGCG was 100 μM, the survival rate of Nalm6 and human lymphocyte decreased by 50% and 25%, respectively [130]. 

## 6. Conclusions

In this paper, the phytochemical constituents and pharmacological activities of green tea were systematically and comprehensively reviewed. Catechin, caffeine, theanine, tea polysaccharide, and other chemical components in green tea have pharmacological activities and health care functions, such as antioxidant, anti-tumor, hypoglycemic, and so on. As a natural antioxidant, tea polyphenols have been widely used in the food industry and cosmetics. In addition, the catechins in green tea also play an important role in the prevention and treatment of diabetes, hepatitis, microbial/viral infections, cancer, and skin inflammation. 

In 2006, the FDA approved “Veregen ointment”, a green tea extract external preparation, for clinical use and it has already appeared on the market in the United States. However, the research on the pharmacological activity of green tea is still in the laboratory research stage. Therefore, how to carry out in-depth research on the mechanism of green tea active ingredients and realize the conversion from research to clinical application is still a major challenge facing researchers. Finally, there are few reports on toxicological studies of green tea, mainly related to hepatotoxicity and cytotoxicity. Therefore, toxicity studies are still a potential research area in the future.

## 7. Perspectives

First, there have been numerous studies to prove that many chemical components in green tea have significant pharmacological activities. However, little is known about the relationship between the chemical structure, physicochemical properties of these components, and pharmacological activity. EGCG is the most biologically active catechin in green tea. Chao et al. found that the solubility of EGCG after acetylation (pEGCG) was significantly improved, and activity analysis experiments showed that pEGCG was more effective in preventing neurodegenerative diseases than EGCG [131]. Therefore, in addition to the solubility of the compound, the relationship between its molecular weight, bonding type, functional group distribution, and physiological activity needs to be further explored.

Second, tea polyphenol (TP) is a general term for polyhydroxy phenol compounds contained in green tea. It is the main active ingredient of green tea and has various pharmacological effects such as scavenging free radicals and anti-tumor. However, tea polyphenols have poor lipid solubility, low bioavailability, and are easily oxidized, which limits their application in the pharmaceutical and food industries. At present, some new preparation technologies such as liposome and nanoemulsion have been used to improve the bioavailability of tea polyphenols in vivo [132,133]. These technologies generally have the problems of complicated preparation processes and residual organic solvents. Therefore, based on a comprehensive variety of technologies, it is necessary to introduce new technologies to give full play to the active role of green tea polyphenols in disease prevention and treatment.

Third, China is the world’s largest exporter of green tea. The export volume of green tea accounts for more than 80% of the international market, but the average export price is generally lower than the top five in the world. Therefore, the tea industry should be promoted to form a set of standardized, engineered, and large-scale new industrial models, and ultimately to achieve modernization and internationalization. (1) Standardization of evaluation methods of green tea. Emerging detection technologies, such as electronic eyes, electronic tongues, and electronic noses, are used to detect the color, taste, and aroma of tea soup. It is simple, convenient, low cost, gives reliable results, highly repeatable, and has a broad application prospect in the field of green tea quality assessment. (2) Improvement of quality and safety standards of green tea. Due to the large number and variety of green tea in China, it is unrealistic to uniformly specify the name, quality, and grade of all green tea products under a single standard. Therefore, on the one hand, according to the quality characteristics of different varieties of green tea, other standards in this series can be continuously formulated and supplemented, so that the green tea quality standards can be continuously enriched and improved. On the other hand, it is necessary to continuously strengthen the integration between the Chinese tea standard system and international standards, and the adaptability to the market economy.

## Figures and Tables

**Table 1:** Pharmacological effects of green tea.

Pharmacological Effects	Detail	Extracts/Compounds	Minimal Active Concentration/Dose	In Vitro/In Vivo	Refs.
Antioxidant effects	Inhibiting copper-catalyzed low-density lipoprotein (LDL) lipid peroxidation	Tea polyphenols	0.1 μg/mL	In vitro	[37]
	Scavenging DPPH radicals	EGCG, ECG, EGC, and EC	EC_50_ = 0.03, 0.04, 0.07, and 0.10 mol/mol DPPH, respectively	In vitro	[39]
	Scavenging total oxy-radicals	ECG and EGCG	0.348 ± 0.012, and 0.374 ± 0.020 TOSC/ μM	In vitro	[40]
	Scavenging superoxide radicals	TLPS, TFPS and TSPS	0.5 μg/mL~100 μg/mL	In vitro	[41]
	Increasing the activity of antioxidant enzymes	Green tea extract	125, 625 and 1250 mg/kg (i.g., for four weeks)	In vivo	[42]
	Protecting against hepatotoxicity caused by excess acetaminophen (APAP) in mice	Green tea extract	10 mg/L	In vivo	[43]
Anticancer effects	Inhibiting migration and invasion of tumor cells inhibited the growth of HeLa cells	EGCG	IC_50_ = 57.2%, and 29.3%, (48 h), respectively	In vitro	[46]
	Inhibiting lung and liver metastasis in BALB/c mice with 4T1 tumors	Green tea extract	0.06~0.125 mg/mL, respectively	In vivo	[47]
	Inducing apoptosis in colon cancer cells by modulating Akt, ERK1/2 and p38 MAPK signaling pathways	EGCG	100 or 200 μM	In vitro	[48]
	Inducing apoptosis in ovarian cells	Green tea extract	0.1, 1, 10, 100, and 200 μg/mL, respectively	In vitro	[49]
	Inhibiting the growth of HuH7 cells and HCC cells	EGCG	IC_50_ = 25, 84 μg/mL, respectively	In vitro	[50]
	Inhibiting the growth of androgen-sensitive and androgen-sensitive human prostate cancer cells (PCA)	EGCG	10 µg/mL~80 µg/mL	In vitro	[51]
	Inhibiting the growth of gastric cancer cell line NCI-N87 in a time- and dose-dependent manner	EGCG	0~100 μM	In vitro	[52]
	Inhibiting the growth of oral cancer tumors	Green tea polyphenols	200 mg/kg	In vitro	[54]
	Inhibiting cyclooxygenase-2 in non-small cell lung cancer cells	Green tea polyphenols	0, 10, 20, and 40 μg/mL, respectively	In vitro	[55]
Anti-diabetic effects	Increasing insulin sensitivity in rats	Green tea polyphenols	0.75%	In vivo	[58]
	Improving insulin sensitivity and lipid distribution	Green tea extract	80 mg/kg (i.g., for 12 weeks)	In vivo	[60]
	Improving glucose metabolism	Green tea extract	75 mg/kg (i.g., for 30 days)	In vivo	[61]
	Promoting insulin secretion	The water-soluble polysaccharide 7WA	50 µg/mL~200 µg/mL	In vitro	[62]
	Alleviating maternal diabetes-induced neural tube defects	EGCG	10 µM	In vivo	[66]
Antibacterial effects	Inhibiting Helicobacter pylori activity	EGCG	MIC_90_ = 50~100 μg/ml	In vitro	[67]
	Inhibiting the survival of Mycobacterium tuberculosis in macrophages	EGCG	0~60 μg/mL	In vitro	[68]
	Inhibiting S. aureus activity	EGCG	MIC_90_ = 58 mg/L	In vitro	[70]
	Inhibiting skin pathogens activity	Green tea extract	MIC = 0.156~0.313 mg /mL	In vitro	[71]
	Inhibiting Porphyromonas gingivalis activity	Green tea extract and EGCG	MIC = 250~1000 mg/mL, 125~500 mg/mL, respectively	In vitro	[74]
Antiviral effects	Inhibiting HIV-1 integrase activity	EGCG and GC	IC_50_ = 0.96, 0.56 μmol/L, respectively	In vitro	[77]
	Inhibiting influenza A virus activity	EGCG	EC_50_ = 22~28 μM	In vitro	[79]
	Inhibiting the production of hepatitis B virus (HBV)	Green tea extract	EC_50_ = 5.02, 5.681, 19.8, and 10.76 μg/mL, respectively	In vitro	[80]
	Inactivating the Foodborne viruses, such as human Novo virus (NoV) and hepatitis A virus (HAV)	Green tea extract	10 mg/mL (for 30 min)	In vitro	[81]
	Inhibiting ZIKV entry into host cells	EGCG	Not mentioned	In vitro	[83]
Neuroprotective effects	Improve learning and memory in aged rats through the antioxidant defense	Green tea extract	0.5% (i.g., for 8 weeks)	In vivo	[87]
	Improve memory and hippocampal LTP in AD mice through the dopamine D1/5-PKA pathway	L-theanine	12.5 μM~50 μM	In vivo	[88]
	Neuroprotective effect on neurotoxins in mice and rat	EGCG	Not mentioned	In vivo	[90]
	Protect dopamine neurons in 6-OHDA-treated PD rat models	Green tea polyphenols	450 mg/kg/day	In vitro	[92]
	Improve nerve redox imbalance and mitochondrial dysfunction by regulating circadian rhythm	Green tea polyphenols	10, 20, and 40 µg/mL, respectively	In vitro	[93]
	Reduce neuronal damage after cerebral ischemia	EGCG	50 mg/kg	In vivo	[95]
	Improve the spatial cognitive ability after chronic cerebral hypoperfusion	Green tea polyphenols	400 mg/kg	In vivo	[96]
	Inhibiting HO-1 expression and activating ERK1/2 pathway	Theanine	1 mg/kg	In vivo	[97]
	Reduces (DDT)-induced cell death in dopaminergic SHSY-5Y cells	EGCG	1, 3 and 10 µM	In vitro	[100]
	Protecting against lead-induced brain oxidation and DNA damage in rats	Green tea extract	5 g/L	In vivo	[102]
	Improve cognitive impairment caused by isoflurane by regulating oxidative stress	Green tea polyphenols	25 mg/kg (i.g., for 7 days)	In vivo	[105]
Effects on the immune system	Inhibit the proliferation of spleen T cells in C57BL mice	EGCG	2.5 μM~10 μM	In vivo	[106]
	Inducing regulatory T cells	EGCG	2%, *w*/*v*	In vitro	[107]
	Reduce the mortality of mice with anaphylactic shock induced by compound C48/80	Green tea extract	11, 13, and 15 mg/mL	In vivo	[108]
	Improve T-cell-mediated autoimmune diseases	EGCG	0.3%	In vivo	[109]
Other pharmacological effects	Inhibited the mutagenicity of tobacco in a concentration-dependent manner	Green tea polyphenols	50 mg/plate	In vitro	[111]
	Anti-thyroid effect	Green tea extract	1.25 g%, 2.5 g%, and 5.0 g%, respectively	In vivo	[112]
	Diuretic activity	Green tea extract	100~500 mg/mL	In vivo	[115]
	Bone-protective effect	green tea polyphenols	0.1% or 0.5% concentration	In vivo	[117]
	Anti-protozoal Leishmania effect	EGCG and ECG	IC_50_ = 27.7, 75 μM, respectively	In vitro	[118]

## Data Availability

Not applicable.

## Abbreviations

ROS	reactive oxygen species
MMP-9	matrix metalloprotein-9
TIMP-1	tissue inhibitor of metalloproteinase-1
HT-29	human colorectal adenocarcinoma grade II
Akt	extracellular signal-regulated kinase
ERK1/2	extracellular signal-regulated kinase 1/2
MAPK	mitogen-activated protein kinase
EIA	enzyme linked immunosorbent assay
VEGF	vascular endothelial growth factor
KLF4	kruppel-likefactor 4
4-NQO	4-Nitroquinoline 1-oxide
COX-2	cyclooygenase-2
ANX1	annexin-1
PPAR	peroxisome proliferator-activated receptors
LPL	lipoproteinlipase
GLUT-4	glucose transporter-4
cAMP	cyclic AMP
TACO	tryptophan-aspartate containing coat protein
HSCCC	high-speed counter-current chromatography
LTP	long-term potentiation
PKA	protein kinase A
6-OHDA	6-hydroxydopamine
HO-1	heme oxygenase-1
DDT	dichlorodiphenyl-trichloroethane
IL-2	interleukin 2
p53	protein 53
p38	protein 38
HIV-1	human immunodeficiency virus-1
AD	Alzheimer’s disease
PD	Parkinson’s disease
Bax	BCL2-Associated X Protein
Bcl-2	B-cell lymphoma-2
